# Infratemporal fossa approach: the modified zygomatico-transmandibular approach

**DOI:** 10.1186/s40902-018-0185-x

**Published:** 2019-01-11

**Authors:** Soung Min Kim, Sun Ha Paek, Jong Ho Lee

**Affiliations:** 1 Oral and Maxillofacial Microvascular Reconstruction LAB, Ghana Health Service, Regional Hospital Sunyani, P.O. Box 27, Sunyani, Brong Ahafo Ghana; 2 Department of Oral and Maxillofacial Surgery, Dental Research Institute, Clinical Trial Center and Oral Cancer Center, School of Dentistry, Seoul National University, Seoul, South Korea; 3 Department of Neurosurgery, Hypoxia Ischemia Hypoxia Disease Institute, Cancer Research Institute, Seoul National University Medical College, Seoul, South Korea

**Keywords:** Infratemporal fossa, Infratemporal fossa approach, Zygomatico-transmandibular approach, Lateral skull base dissection, Preferred Reporting Items for Systematic Reviews and Meta-Analyses (PRISMA)

## Abstract

**Background:**

The infratemporal fossa (ITF) is an anatomical lateral skull base space composed by the zygoma, temporal, and the greater wing of the sphenoid bone. Due to its difficult approach, surgical intervention at the ITF has remained a heavy burden to surgeons. The aim of this article is to review basic skull base approaches and ITF structures and to avoid severe complications based on the accurate surgical knowledge.

**Methods:**

A search of the recent literature using MEDLINE (PubMed), Embase, Cochrane Library, and other online tools was executed using the following keyword combinations: infratemporal fossa, subtemporal fossa, transzygomatic approach, orbitozygomatic approach, transmaxillary approach, facial translocation approach, midface degloving, zygomatico-transmandibular approach, and lateral skull base. Aside from our Preferred Reporting Items for Systematic Reviews and Meta-Analyses (PRISMA) trial, there have been very few randomized controlled trials. The search data for this review are summarized based on the authors’ diverse clinical experiences.

**Results:**

We divided our results based on representative skull base approaches and the anatomy of the ITF. Basic approaches to the ITF include endoscopic endonasal, transzygomatic, orbitozygomatic, zygomatico-transmandibular, transmaxillary, facial translocation, and the midfacial degloving approach. The borders and inner structures of the ITF (with basic lateral skull base dissection schemes) are summarized, and the modified zygomatico-transmandibular approach (ZTMA) is described in detail.

**Conclusions:**

An anatomical basic knowledge would be required for the appropriate management of the ITF pathology for diverse specialized doctors, including maxillofacial, plastic, and vascular surgeons. The ITF approach, in conjunction with the application of microsurgical techniques and improved perioperative care, has permitted significant advances and successful curative outcomes for patients having malignancy in ITF.

## Background

Malignant tumors of the infratemporal fossa (ITF) usually originate from the paranasal sinuses, oral cavity, or the skull base structures. Although the incidence of ITF malignancy is less than 3% of all head and neck tumors, a surgical management is the only treatment for complete control [1–3]. Furthermore, a neurosurgeon and a maxillofacial (plastic or head and neck surgeon) must collaborate to accomplish a good prognosis without complications.

The treatment of skull base tumors is a multidisciplinary work composed of plastic and reconstructive surgeons, anesthesiologists, radiologists, rehabilitation specialist, and neurosurgeon. Additionally, medical and radiotherapeutic oncologists are also important for their chemotherapy combined with radiation therapy pre- or postoperatively.

In this review article, we focus on representative skull base approaches and the complex anatomy of the ITF by summarizing its borders, inner structures, and lateral skull dissection techniques. This article also describes the modified zygomatico-transmandibular approach (ZTMA), which is presented in detail from the maxillofacial surgeon’s point of view.

## Main text

A search of the recent literature using MEDLINE (PubMed), Embase, Cochrane Library, and other online tools was executed with the following keyword combinations: infratemporal fossa, subtemporal fossa, transzygomatic approach (TZA), orbitozygomatic approach (OZA), transmaxillary approach (TMA), facial translocation approach (FTA), midface degloving approach (MDA), zygomatico-transmandibular approach (ZTMA), and lateral skull base. The search data were summarized based on the authors’ diverse clinical experiences. A statement of ethics approval was provided by the Department of Oral and Maxillofacial Surgery at Seoul National University Dental Hospital, with the approval of the Institutional Review Board of Seoul National University (S-D20170024).

We approached to summary variations in infratemporal skull base approaches, and the clinical anatomy of the ITF.

### Representative skull base approaches

The infratemporal fossa approach (ITFA) is one of the several approaches to the skull base, including maxillary swing, midfacial degloving, facial translocation, endonasal transsphenoidal, subfrontal interhemispheric, orbitozygomatic, pterional, subtemporal, middle cranial fossa, translabyrinthine, transcochlear, retrolabyrinthine presigmoid, retrosigmoid suboccipital, far lateral transcondylar, transcervical, and transpalatal [3–5].

#### Endoscopic endonasal approach

**Fig. 1:**
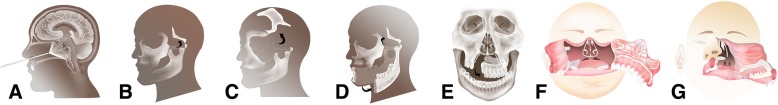
Schematic drawings of representative skull base approaches showing the endoscopic endonasal approach (**a**), the transzygomatic approach (**b**), the orbitozygomatic approach (**c**), the zygomatico-transmandibular approach (**d**), the transmaxillary approach (**e**), the facial translocation approach (**f**), and the midfacial degloving approach (**g**)

Compared with extensive transcranial approaches for the sinonasal or nasopharyngeal malignancies, endoscopic endonasal approach (EEA) was developed as an alternative to the middle and posterior cerebral fossae [6]. EEA could provide a less invasive approach to the access difficulty lesions especially including the pterygopalatine fossa (PPF) and reduce the morbidity rate of transcranial surgical approaches [7]. EEA is recommended as a minimally invasive approach to the PPF which has rich vasculonervous contents between intra- and extracranial compartments. Zero to seventy-five-degree lens endoscopes are recommended for optimal visualization at different angles (Fig. 1a), and the middle meatal EEA could be further divided into transpalatine approach, transantral approach, and the endonasal inferior turbinectomy [8].

#### Transzygomatic approach

For the approach to the anterior temporal dura and middle fossa, TZA could be considered by vertical sectioning of zygomatic arch and by the fronto-temporo-sphenoidal craniotomy (Fig. 1b). TZA is appropriate for the approach to the interpeduncular cistern region by combining with pre- and transtemporal access [9] and also to the superior portion of the ITF, PPF, and orbit [10, 11].

#### Orbitozygomatic approach

OZA could be chosen for the wide exposure of lateral skull base, middle cranial fossa, basilar apex, and ITF [12]. Pterion approach combined with supraorbital craniotomy has been a routine neurosurgical practice by osteotomy of the orbital walls (Fig. 1c). The orbital bar could be preserved for keeping its original position after fully exposing the superior orbital fissure (SOF) and neurovascular structures.

#### Zygomatico-transmandibular approach

The ZTMA is a modified approach to lateral skull base surgery. It combines the TZA and the OZA, adding a mandibulotomy procedure (Fig. 1d). For most ITF tumors, coronoidectomy should be included to fully expose masticatory muscles, related vessels, and cranial nerves. The transmandibular approach (TMA) is a popular way to excise maxillary sinus tumors, especially those extending to the PPG and ITF [13]. Mandibular swing can be relocated using titanium miniplates before the mandibulotomy procedure. The mandibulotomy procedure can be performed prior to zygoma or orbital osteotomy for convenience [14].

#### Transmaxillary approach

TMA directly exposes the anterior and lateral skull base including ITF. Several important anatomies including mandibular branch (V3), PPF, maxillary artery (MA), lateral pterygoid muscle (LPM), foramen rotundum, and foramen ovale are exposed sequentially [15] in this TMA. Although a paralateronasal incision is inevitably made (Fig. 1e), TMA is an essential way to the extensive pathology in the retroantral, pterygomaxillary fissure (PMF), and PPF [16].

#### Facial translocation approach

FTA is a somewhat rare approach, but often chosen for extensive tumors involving nasopharynx, nasal cavity, posterior maxillary sinus, and ITF. This unesthetic approach could be chosen in these order, such as subtemporal, preauricular, infratemporal, maxillary swing, TMA, and finally FTA [17]. Excluding a standard facial translocation, more limited osteotomies including a unilateral or bilateral medial translocation are indicated for smaller tumors [18]. An extended medial facial translocation combined with midfacial degloving can minimize the sequelae of the FTA (Fig. 1e).

#### Midfacial degloving approach

From 1992 onward, MDA has gained popularity and could be effective in removing a tumor without late complications. Closed rhinoplasty incisions with direct opening of nasal cavity, paranasal sinus, and anterior skull base structures are essential for the MDA (Fig. 1f). For the complete resection of an angiofibroma on the skull base, the combined transnasal-transpalatal approach is effective. This is especially true for the complete removal of tumors with severely aggressive bleeding tendencies. Such tumors are often confined to the nasal cavity and nasopharynx, with ITF extensions [19].

### Infratemporal fossa anatomy

The complex lateral skull base and its anatomical structures are affected in various ways by benign and malignant tumors. Although each clinician has preferred approaches, diverse approaches should be considered. A basic anatomical knowledge of the ITF is essential to choose the appropriate approach for each pathologic entity [10].

#### Borders and inner structures of the infratemporal fossa

**Fig. 2:**
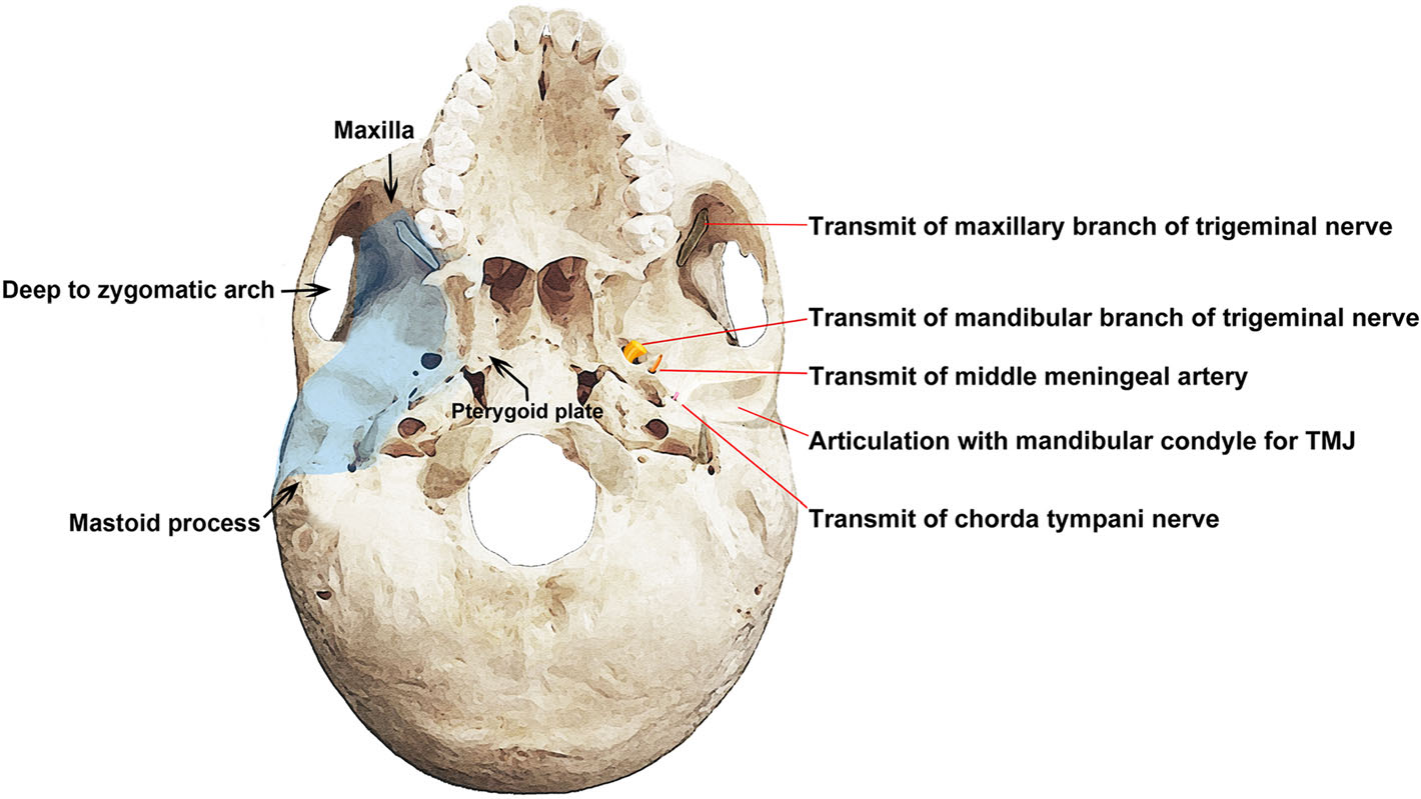
Lateral skull base showing the boundary (left) and contents of the infratemporal fossa (right)

The basic ITF space is inferior from the zygoma, lateral to the mandibular ramus, medial to the lateral pterygoid plate, posterior to the tympanic plate with mastoid and styloid processes, and anterior to the posterior maxillary wall (Fig. 2). Anteriorly, ITF connects with maxillary tuberosity and PMF and the inferior orbital fissure (IOF) can be seen with hamulus inferiorly. Posteriorly, the infratemporal surface of the sphenoidal greater wing is connected with the temporal squamous portion, and the inferior border of the ITF exists where the medial pterygoid muscle (MPM) attaches to the mandibular angle (Fig. 2).

The contents of the ITF are mainly the muscles of mastication (including the temporalis, masseter, lateral, and medial pterygoid muscles), vessels (including MA with veins and the pterygoid plexus of veins), nerves (including the mandibular, lingual, and otic ganglia), and the temporomandibular joint (TMJ).

LPM has a superior head with infratemporal crest and surface initiation, the anterior capsule of TMJ insertion, an inferior head with a lateral surface for pterygoid plate initiation, and the pterygoid fovea below the mandibular head and disc. The main action of the LPM is protruding the jaw and opening the mouth. MPM has a deep head on the lateral pterygoid plate and a smaller superficial head. The main action of the MPM is pulling the mandible upwards, forwards, and medially and closing the mouth during chewing. The LPM and MPM contain venous plexuses that connect with facial veins.

The MA, or internal maxillary artery (IMA), is composed of three parts according to its course.

Branches of the first mandibular part of the MA sit between the initial mandibular part and the posterior part of LPM. They include the auricular artery deep to the external acoustic meatus, the tympanic artery anterior to the tympanic membrane, the middle meningeal artery (MMA) to the dura mater (with calvaria), the accessory meningeal artery to the cranial cavity, and the alveolar artery inferior to the mandibular gingiva and teeth. The second part of the MA is the pterygoid portion between LPM and PPF. It mainly serves the masticatory muscles and includes the masseteric artery, deep temporal artery, pterygoid branch, and buccal artery. The third pterygopalatine part comprises a short course in the PPF, from the sphenopalatine artery to the sphenopalatine foramen. It includes the posterior superior alveolar, infraorbital, descending palatine, sphenopalatine artery, and artery of pterygoid canal.

The deep veins of the face can be divided into the maxillary vein and pterygoid plexus. These are counterpart to the superficial veins of the face (i.e., the facial, superficial temporal, posterior auricular, occipital, and retromandibular veins). The maxillary vein courses with the first division of the MA, starting from the posterior part of the pterygoid plexus and continuous with the superficial temporal and retromandibular vein. The pterygoid plexus forms the main venous bundles in the ITF. It is located between the LPM and MPM and between the temporal muscle and LPM. The pterygoid plexus communicates with the anterior facial vein and cavernous sinus through the foramen Vesalius, foramen lacerum, and foramen ovale.

Nerves within the ITF are divided into three groups: branches from the trunk (including the spinous nerve and medial pterygoid nerve), anterior branches (including the buccal, masseteric, deep temporal, and lateral pterygoid nerve), and posterior branches (including the auriculotemporal, lingual, and inferior alveolar nerve). The V3 descends through the foramen ovale into the ITF and divides into sensory and motor branches. These comprise the auriculotemporal, inferior alveolar, lingual, and buccal nerves. Branches also supply the four muscles of mastication, but not the buccinator (which is supplied by the facial nerve). The chorda tympani nerve (CTN) carries taste fibers from the anterior two thirds of the tongue and parasympathetic innervation to the submandibular and sublingual glands. It originates from the facial nerve in the temporal region and passes anteriorly to enter in the middle ear, where it is separated from the tympanic membrane by the handle of malleus. It enters the ITF and descends medial to the spine of sphenoid and then to the LPM.

#### Lateral skull base dissection

Basic lateral skull dissection could be proceeded as follows: (1) check and confirm the origin and insertion of the masseter muscle; (2) retract the cutting zygomatic arch and inserted masseter muscle in a downward: (3) check and confirm the insertion of the temporal muscle to the coronoid process; (4) confirm the masseteric nerve and vessels at the mandibular notch; (5) carefully cut the coronoid process without causing soft tissue injury by approaching antero-inferiorly from the mandibular notch. Inspect the coronoid process with the attached temporalis and remove the temporal muscle after locating the anterior and posterior deep temporal nerve and vessels; (6) after marking the horizontal line on the external surface of the lingual nerve, cut without injuring the mandibular foramen or inferior alveolar nerve and vessels; (7) separate the ramus from the insertion of the periosteal elevator to the inside of the mandibular neck; and (8) after removal of this portion of the ramus, the contents of the ITF will be exposed. Two significant differences, such as the short distance between mandible and ITF, and the more distant location of facial nerve from the mandible, could be considered in the pediatrics [10, 20, 21].

### Detailed procedures of zygomatico-transmandibular approach

In spite of disagreement among some clinicians regarding the exact limits of the ITF and/or recommended surgical approaches, we have described the modified ZTMA here because of its versatility. It is suitable for tumors originating from the ITF, tumors originating from the greater wing of the anterior temporal base or sphenoid bone, and tumors spreading to the ITF. Although the safe resection of tympanic bone or the safe control of the infratemporal facial nerve (or jugular bulb) are not feasible, most ITF tumors in the oral and maxillofacial region can be removed by this conventional approach.

#### Incision with facial flap elevation

**Fig. 3:**
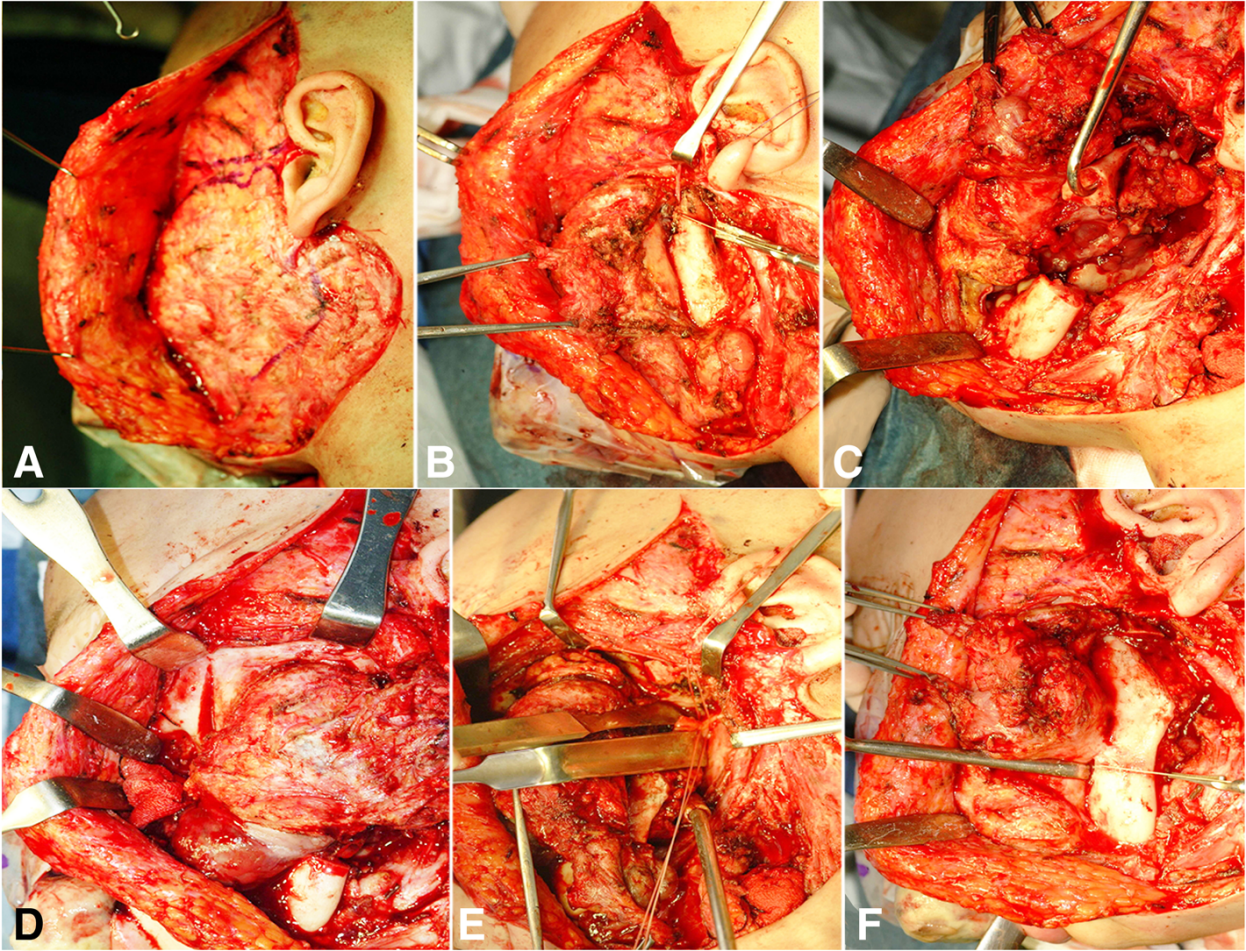
Surgical procedures of the modified zygomatico-transmandibular approach to the infratemporal fossa, showing the incision with facial flap elevation (**a**), exposure and isolation of the facial nerve with mandibulotomy (**b**), direct exposure of the infratemporal fossa (**c**), mass dissection by orbito-zygomectomy with temporal muscle dissection (**d**), identification of the internal jugular vein, internal maxillary artery, ligation (**e**), and mass resection with temporal craniotomy (**f**)

**Fig. 4:**
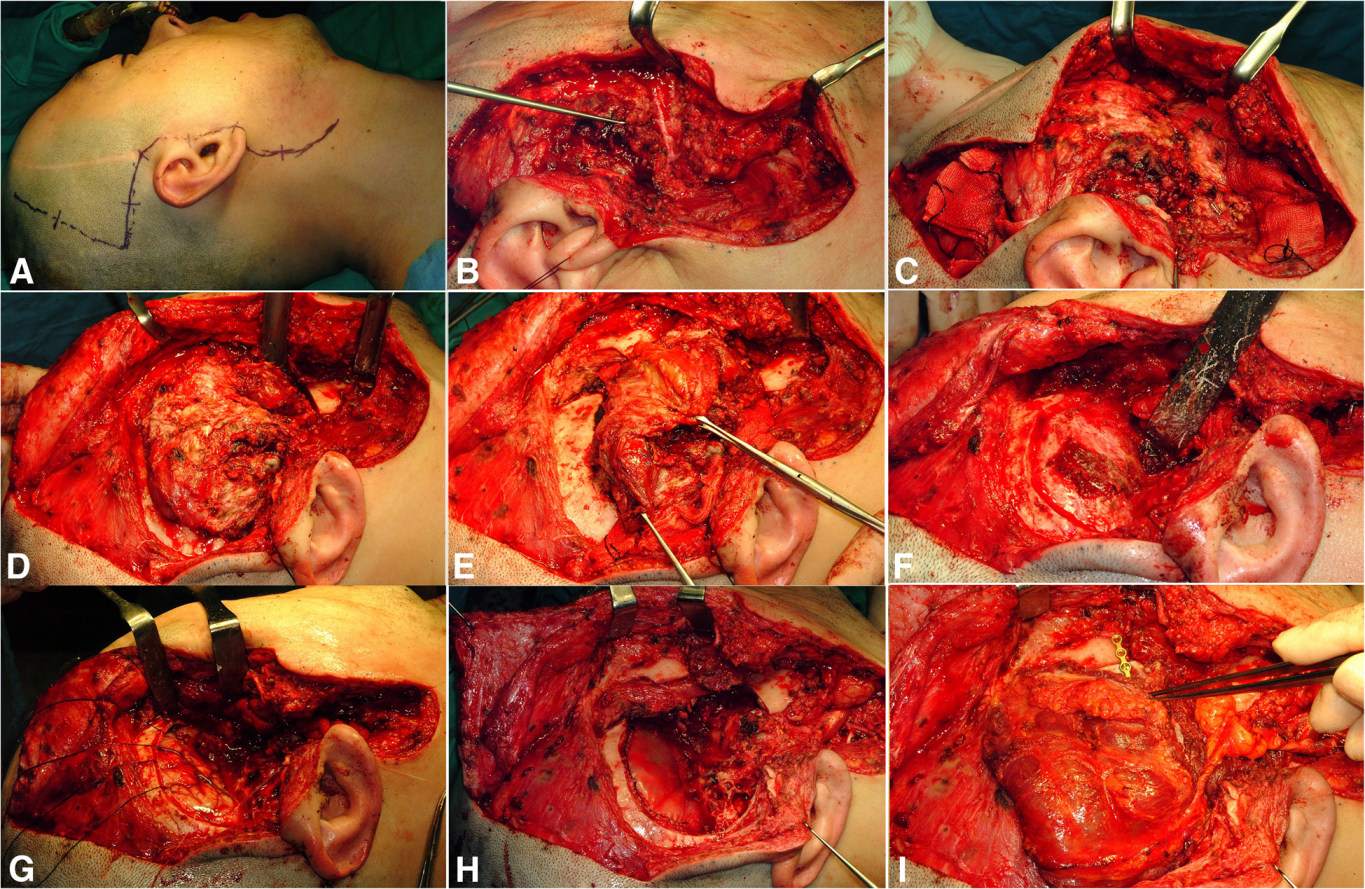
The modified zygomatico-transmandibular approach to the infratemporal fossa with latissimus dorsi muscular free flap reconstruction, showing the incision line (**a**), mobilization of the parotid gland (**b**), pathologic mass exposure by orbito-zygomectomy (**c**) and coronoidectomy (**d**), whole mass removal (**e**), the temporal or skull base craniotomy state (**f**), and dura mater repair with latissimus dorsi free flap reconstruction (**g** to **i**)

**Fig. 5:**
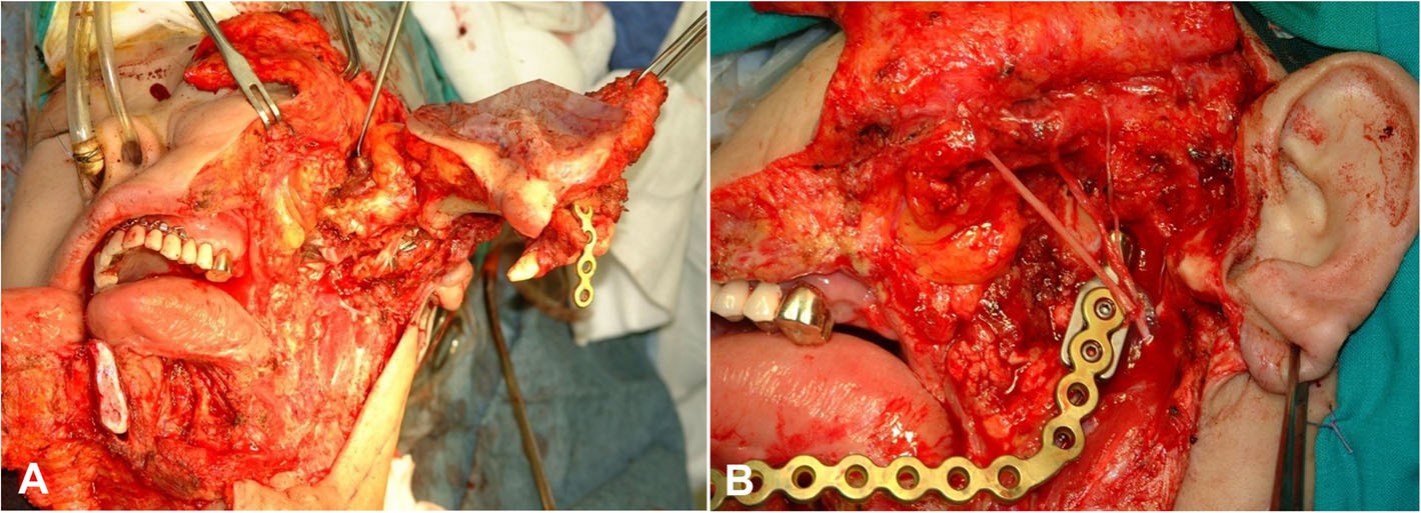
Surgical images showing the zygomatico-transmandibular approach to the lateral skull base (**a**) and its repositioned state, with reconstruction plate and sural nerve graft to the facial nerve trunk (**b**)

The initial incision could be adjusted by combining a hemicoronal or submental-submandibular to the conventional parotidectomy incision for the lateral transparotid approach. The anterior facial flap elevation plane is similar to the normal facial lifting that occurs when the facial nerve trunk is not cut, and the parietomassetric fascia is dissected outward (Fig. 3a and Fig. 4a). In the upper part of the zygoma, the frontal, and zygomatic branches of the facial nerve could be seen by delamination of the areolar tissue layer outside the temporal fascia. If the parotid gland should be removed, an additional peripheral nerve graft could be considered (Fig. 5b).

During the transfacial dissection approach, the dissection proceeds along the deep part of the transfacial layer so as not to damage the frontal branch. In the lower part of the preauricular region, the dissection is performed like a facial lifting along the parotidial-masseteric fascia. The incision is usually similar to the radical neck dissection incision, connecting the mastoid process and the hyoid bone. The dissection of the scalp flap extends to the superior orbital rim along the subpericranial plane. The cheek area extends to the parotid gland, and the subclavian incision is usually sufficient for the mental foramen.

#### Mobilizing the parotid gland

Full mobilization of the entire parotid gland is necessary for localization of facial nerve main trunk. At first, the parotid sheath near the zygoma is removed from the external auditory meatus. The parotid gland is separated from the tragal cartilage and bony ear canal. The mastoid-leading edge and the ear lobule are separated. Then, sharp dissection of the sternocleidomastoid (SCM) muscle, adherent fascia (attached to the parotid tail), and sternomandibular ligaments (connected to the mandibular angle) can be performed. At this point, the anterior branch of the external jugular vein and the greater auricular nerve should be cut. If possible, the posterior branch should be preserved for ear lobe sensation. If the external meatus cartilage is further dissected from the parotid capsule, the bony resistance of the styloid process can be felt. When the tail of the parotid is pulled all the way to the outside, the retromandibular vein is seen from the parotid gland. At this point, bleeding due to congestion should be avoided. The whole cleavage should be retained, as this may help with access to the facial nerve (Fig. 3a and Fig. 4b).

#### Facial nerve exposure and main trunk transection

For identification and exposure of facial nerve main trunk, the posterior digastric muscle, stylohyoid muscle, and stylopharyngeus muscle can be seen by anterior gentle traction of the mobilized parotid gland. The pointer cartilage tip and the vaginal process of the styloid process are visible during further dissection (Fig. 3b). In most cases, the nerve trunk is exposed by the tragal cartilage pointer method. A tunnel is made 5 to 10 mm into the parotid gland by inserting a medium-sized Kelly or mixer clamp along the lateral margin of the nerve. The parotid gland is incised to isolate each branch.

#### Orbitozygomatic osteotomy

After exposing the zygomatic body, the periosteum is stripped and zygomatic cortex is removed by osteotomy at the zygomatic root for the posterior part, the zygomaticofrontal suture for the upper part, and the zygomaticomaxillary buttress. This is on the zygomaticofacial nerve level from the inside. The zygomatic arch can be separated from the masseter muscle by transection with electrocautery or can be moved downward with the masseter muscle attached, to expose the ITF (Fig. 3c, Fig. 4c, Fig. 5a).

In order to confirm the IOF, orbitozygomatic osteotomy is essential. After the periorbit is elevated from the outer or lower wall of the orbit, the tip of the reciprocating saw is positioned in the outermost part of the IOF from the lower part of the orbit. Osteotomy should be performed along the malar eminence. A beveled or V-shaped bone cut is performed using a reciprocating saw to increase the exposure and return the bone to its original position after the operation. If the tumor mass does not involve the temporal bone or the petrous portion of the internal carotid artery (ICA), this level of osteotomy is sufficient for exposure to the infratemporal skull base. However, if removal of the petrous portion of the ICA is required, the glenoid fossa should be removed together with osteotomized bony segments, and temporal craniotomy may be performed to expose the glenoid fossa. TMJ capsule can be freed from the fossa and lowered for full exposure, but the capsule and meniscus should be preserved if possible. If additional exposure is required, the condyle can be removed from the sigmoid notch. A V-shaped bone cut can be performed using a reciprocating saw to remove the outer two thirds of the glenoid fossa. By thus removing the glenoid fossa, the ICA inside the glenoid fossa can be protected.

#### Temporal muscle dissection with coronoidectomy

The pericranium-attached temporal muscle is elevated from the temporal fossa by the remaining 2–3 mm fascia on the muscular margin, for its relocation during closing reconstruction. Inferior observation of the temporalis is recommended until full exposure of the infratemporal crest is achieved. The soft tissues of the sigmoid notch should be protected to prevent IMA injury inside of the mandibular ramus, and the coronoid process should be removed or fractured to increase the inferior rotation arc of the temporal muscle. Wide dissection on the soft tissues of the infratemporal skull base is not recommended due to continuous venous bleeding in the pterygoid plexus. Subtemporal craniotomy can be used to confirm the neural and vascular foramina (Fig. 3c and Fig. 4d).

#### Mandibulotomy and/or partial mandibulectomy

Although mandibulotomy can give a wide additional exposure, partial mandibulectomy should be executed (for a negative tumor margin) in the case of invasive mandibular malignancy. For preserving the mandibular condyle, mandibular cutting near the third molar should be done after exposure of the condylar head. The joint capsule and inner tissue can be protected with a curved periosteal elevator. Complete detachment of the masseter and medial pterygoid muscles from the ramus should be followed. The resected mandible can be retracted anteriorly, and ligations of inferior alveolar nerves and vessels on the lingual course performed, for full exposure of the anatomical contents of the ITF (Fig. 3d, Fig. 4e, and Fig. 5a).

#### Identification of the ICA, IJV, and IMA locations

Further lateral dissection of the SCM muscle through the caudal limb of the initial incision line can expose the carotid sheath with the internal, common, and external carotid artery and internal jugular vein (IJV). Cranial nerves, including the vagus, spinal accessory, and hypoglossal, should be confirmed and preserved. IMA in the ITF and its related branches could be also identified (Fig. 3e).

#### Mass resection with temporal or skull base craniotomy

Additional bone removal can occur with a bone rongeur after temporal craniotomy. Using the curved line of the lateral pterygoid plate, the foramen ovale, foramen spinosum, and sphenoidal spine are found posteriorly. If needed, cauterization of the MMA with bipolar electrocautery and surgical packing for the purpose of bleeding control at the venous communication on the foramen ovale should be done. If complete detachment of the petrous portion of the ICA is needed, V3 can be cut on the foramen ovale. The whole bone and soft tumor mass can be removed by the appropriate evaluation of the sphenoid sinus after resection of the bone between maxillary branch (V2) and V3 (Fig. 3f and Fig. 4f).

#### Dura mater repair with closing reconstruction

Exposed dura mater should be closed by water-tight suture combined with lyophilized dura or SCM muscle graft. All upper aerodigestive tract defects related to tumor resection should also be closed. If the temporalis muscle with its vascular supply is intact, trigeminal nerve transposition is beneficial for covering the ICA and for dead space closure. Repositioning of the resected orbitozygomatic bone with titanium microplates or 2–0 braided nylon sutures should be considered first, only excluding the intentional zygoma removal for avoiding any compression on flap closure. TMJ reconstruction with repositioning of the resected mandibular condyle should also be considered in the case of exposed petrous ICA (Fig. 4g and i).

#### Prevention and management of postoperative complications

ITF should be selected according to age, gender, facial growth or shape, biological characteristics of pathology, and surgeon’s experience. Any approach should strive for the complete resection of pathology and a careful consideration of postoperative consequences (both esthetic and functional). Minimal but sufficient exposure for the complete resection of pathology is a high priority, along with minimizing the risk to cranial nerves (such as the trigeminal, facial, hypoglossal, vagus, and spinal accessory nerves).

A focus on the prevention and management of postoperative complications is important, and we have summarized the following recommendations: (1) careful bony cutting is needed to avoid hearing loss due to cochlear injury. Comparing the strong marrow bone near the cochlea and labyrinth to the other marrow bone is a useful technique. (2) During dissection to the petrous bone, a close approach near the cartilaginous ear canal can prevent facial nerve injury, and no over-retraction of the greater superficial petrosal nerve should be kept. (3) A damaged ICA should be directly restored after temporary clipping. To avoid ICA injury, only a diamond drill is recommended when removing bone near the ICA. Where the ICA courses through the temporal bone, the periosteal layer surrounds and protects it. Two or three sympathetic nerves run between the ICA and periosteal layer. Therefore, it is necessary to preserve the periosteal layer as much as possible during ICA dissection. (4) For the prevention of trigeminal ganglion and root injury, excessive traction or manipulation should be avoided. (5) In the case of dura injury, CSF may leak into the skin or eustachian tube. If damage occurs, it should be restored with a fat or temporoparietal fascial flap. The cartilage portion of the eustachian tube should be carefully closed. If necessary, head elevation and spinal drainage should be considered, with prophylactic antibiotics administration.

## Conclusions

We have analyzed the following surgical approaches to ITF: incision with facial flap elevation; mobilization of the parotid gland; facial nerve exposure and main trunk transaction; orbitozygomatic osteotomy; temporal muscle dissection with coronoidectomy, mandibulotomy, and/or partial mandibulectomy; identification of the ICA, IJV, and IMA locations; mass resection with temporal or skull base craniotomy; and dura mater repair with closing reconstruction. This ZTMA is versatile and often recommended based on our previous surgical experiences. The ITF approach, in conjunction with the application of microsurgical techniques and improved perioperative care, has permitted significant advances in the successful curative outcomes of patients with extended malignant pathology of the lateral skull base.

## Abbreviations

FTA: Facial translocation approach
ICA: Internal carotid artery
IJV: Internal jugular vein
IMA: Internal maxillary artery
IOF: Inferior orbital fissure
ITF: Infratemporal fossa
ITFA: Infratemporal fossa approach
LPM: Lateral pterygoid muscle
MA: Maxillary artery
MDA: Midface degloving approach
MPM: Medial pterygoid muscle
OZA: Orbitozygomatic approach
PMF: Pterygomaxillary fissure
PPF: Pterygopalatine fossa
SCM: Sternocleidomastoid muscle
TMA: Transmandibular approach
TMA: Transmaxillary approach
TZA: Transzygomatic approach
V3: Mandibular branch
V2: Maxillary branch
ZTMA: Zygomatico-transmandibular approach
